# Altered serum levels of IL-36 cytokines (IL-36α, IL-36β, IL-36γ, and IL-36Ra) and their potential roles in Guillain-Barré syndrome

**DOI:** 10.1097/MD.0000000000023832

**Published:** 2020-12-24

**Authors:** Zhikang Zhao, Rui Zhang, Xinxin Gao, Hui Li, Hongbo Liu

**Affiliations:** a Department of Emergency; b Department of Neurology, The First Affiliated Hospital of Zhengzhou University, Zhengzhou 450052, China.

**Keywords:** cytokines, Guillain-Barré syndrome, interleukin-36, serum

## Abstract

Guillain-Barré syndrome (GBS) is an acute autoimmune neurological disorder mainly involving the peripheral nerves. Currently, various cytokines have been shown to be involved in the pathogenesis of GBS. Because of their similar biological structures, interleukin (IL)-36α, IL-36β, IL-36γ, and IL-36 receptor antagonist (Ra) were all renamed and collectively called IL-36 cytokines. The roles of IL-36 cytokines in GBS currently remain unclear.

Forty-two patients with GBS and 32 healthy volunteers were included in our study. Serum IL-36α, β, γ, and interleukin-36 receptor antagonist (IL-36Ra) levels of patients with GBS in the acute and remission phases and healthy volunteers were measured by enzyme-linked immunosorbent assay (ELISA). In addition, we examined the serum levels of other inflammatory factors that have been shown to be involved in GBS pathogenesis, represented by IL-17 and tumor necrosis factor-α (TNF-α). Furthermore, the correlations between the serum levels of IL-36 cytokines and different clinical data or the serum levels of other inflammatory factors in GBS patients were analyzed.

Significantly higher serum IL-36α and IL-36γ levels were measured in the acute phase than in the remission phase and in healthy control (HC) subjects (*P* < .05), while lower serum IL-36Ra levels were measured in the acute phase than in the remission phase and in HC subjects (*P* < .05). Serum IL-36α and IL-36γ levels were positively correlated with GBS disability scale scores (GDSs), while serum IL-36Ra levels were negatively correlated with GDSs. Correlation analyses among inflammatory factors showed that serum IL-36α and IL-36γ levels in GBS patients were positively correlated with serum IL-17 and TNF-α levels, while serum IL-36Ra levels were negatively correlated with the levels of these 2 inflammatory factors. Similar results were observed in cerebrospinal fluid (CSF), IL-36α and IL-36γ levels in CSF were positively correlated with GDSs, while IL-36Ra levels in CSF were negatively correlated with GDSs. Additionally, the serum and CSF levels of IL-36α and IL-36γ in the axonal subtype of GBS patients were higher than those in the demyelination subtype.

Based on our findings, IL-36 cytokines may be involved in the pathogenesis of GBS and some of these cytokines may help predict the disease severity and other clinical characteristics of GBS.

## 1 Introduction

Guillain-Barré syndrome (GBS) is an acute autoimmune neurological disorder that mainly involves the peripheral nerves. It is clinically characterized by limb weakness, weakening, or disappearance of tendon reflexes and progression over several weeks followed by gradual recovery.^[1,2]^ Originally, nerve demyelination was considered the main pathological mechanism of GBS. However, many patients with pathology limited to axonal degeneration were identified in subsequent studies, and these changes were associated with some special antibodies against gangliosides, such as GM1, GD1a, and GQ1b, particularly after *Campylobacter jejuni* (CJ) infection.^[3]^ Currently, GBS is broadly divided into 2 main subtypes: the demyelination subtype and the axonal subtype. The demyelination subtype mainly refers to acute inflammatory demyelinating polyneuropathy (AIDP), which is more common in Western Europe and the United States. The axonal subtypes, consist of 2 common variants: acute motor axonal neuropathy (AMAN) and acute motor and sensory axonal neuropathy (AMSAN), which are associated with prominent axonal injuries and are relatively more prevalent in China, Japan, and Mexico.^[4,5]^ Currently, the exact etiology of GBS still remains unclear. It may be related to a variety of factors such as microbial infections, host susceptibilities, and a disruption in the immune balance.^[6–9]^

Cytokines often bridge components of the immune system and play crucial roles in initiating, propagating, or regulating autoimmune injuries. According to recent studies, various cytokines, such as tumor necrosis factor-α (TNF-α), IL-17, IL-22, IL-27, and CXCL10, are involved in the pathogenesis of GBS.^[10,11]^ The IL-36 family is a very interesting group of newly named cytokines derived from the IL-1 cytokine superfamily, including 3 agonists (IL-36α, β, and γ) and 1 natural receptor antagonist (interleukin-36 receptor antagonist (IL-36Ra)).^[12]^ Because of their similar biological structures and shared receptor (IL-36R), these cytokines were eventually collectively referred to as IL-36 cytokines.^[13,14]^ IL-36 agonists play proinflammatory roles by binding to IL-36R and activating the downstream transcription factor nuclear factor-*κ*B (NF-*κ*B) and mitogen-activated protein kinase (MAPK) signaling pathway.^[15]^ Conversely, IL-36Ra suppresses inflammatory responses by competitively binding to IL-36R and inhibiting downstream signaling.^[16]^ IL-36 cytokines are expressed in many tissues and cells, such as the brain, skin, lungs, gut, dendritic cells (DCs), macrophages, and lymphocytes.^[13,17,18]^ Recent studies have indicated that IL-36 cytokines may be involved in a variety of inflammatory diseases, such as rheumatoid arthritis, psoriasis, and immune thrombocytopenia.^[14,19–21]^ However, the roles of IL-36 cytokines in GBS currently remain unclear.

In our study, we determined the serum levels of IL-36 cytokines in patients with GBS during different phases and healthy controls (HCs). Meanwhile, we explored the potential relationships of serum IL-36 cytokines levels with clinical parameters and other inflammatory factors in patients with GBS.

## 2 Materials and methods

### 2.1 Study subjects

All subjects were recruited from the Department of Neurology, at the First Affiliated Hospital of Zhengzhou University, China. We tried to include as many suitable patients as possible, and 65 patients with GBS were recruited from July 2016 to August 2019. These patients were diagnosed according to the 1990 Asbury and Cornblath criteria. The exclusion criteria included recent infectious diseases, other autoimmune diseases, malignant neoplasms, and severe liver and kidney dysfunction. If these patients met one or more of the exclusion criteria or failed to complete follow-up, their data were excluded. Finally, 42 patients were included in this study and their serum samples were collected in different phases. Additionally, 32 age- and sex-matched healthy volunteers were included as the HCs. The protocols were performed in accordance with the Declaration of Helsinki and approved by the Ethics Committee of the First Affiliated Hospital of Zhengzhou University. Prior to participation, written informed consent was obtained from all participants.

### 2.2 Clinical assessment

Demographic features, clinical symptoms, electrophysiology, serologic testing, GBS disability scale scores (GDSs), cerebrospinal fluid (CSF) parameters, and therapeutic data were collected. Two neurologists with relevant qualifications independently evaluated GDSs to reflect the disease severity of patients with GBS. Serum samples were collected from patients with GBS in the acute phase at 9 ± 3 days from disease onset prior to treatment with intravenous immunoglobulin (IVIG) or plasma exchange (PE). Serum samples were collected in the remission phase at 1 to 3 months after preliminary recovery. Because most healthy volunteers and patients in the remission phase were reluctant to undergo lumbar puncture, we only collected CSF samples in the acute phase before immunotherapy. The levels of protein in CSF were measured using immunoturbidimetry and the white blood cell (WBC) counts in CSF were determined using the manual microscopic counting method. All acquired samples were collected and stored at −80°C until use.

### 2.3 Measurement of cytokine levels

The levels of IL-36α, IL-36β, IL-36γ, IL-36Ra, IL-17, and TNF-α were measured using commercially available human IL-36α, IL-36β, IL-36γ, IL-36Ra, IL-17, and TNF-α enzyme-linked immunosorbent assay (ELISA) kits provided by Shanghai Enzyme-linked Biotechnology Co., Ltd. (Shanghai, China), according to the manufacturer's instructions. The absorbance values of the corresponding substrate were recorded at 450 nm. The levels of these cytokines were calculated from standard curves, and the lowest detectable levels were 10.0 pg/ml, 10.0 pg/ml, 10.0 pg/ml, 8.5 pg/ml, 12.5 pg/ml, and 12.5 pg/ml, respectively.

### 2.4 Statistical analyses

Data are presented as means ± standard deviations or medians (P25, P75). Differences in variables were analyzed using paired or unpaired *t* tests. Pearson's or Spearman's correlation coefficients were calculated to analyze correlations. A two-tailed *P*-value was considered statistically significant at <.05. SPSS 24.0 software (SPSS, IBM, West Grove) was used for statistical analyses.

## 3 Results

### 3.1 Demographic and clinical characteristics of study subjects

The demographic and clinical features of the 42 patients with GBS included in this study are displayed in Table 1. No significant differences were observed in the age at sample collection or gender ratio between the patients with GBS and HCs (*P* > .05). Clinical subtypes are divided into demyelination and axonal subtype.

**Table 1:** Demographic and clinical characteristics of Guillain-Barré syndrome patients and healthy controls.

Clinical characteristics	GBS (n = 42)	HC (n = 32)
Age (yr)	38.86 ± 12.52	38.97 ± 11.65
Gender (F:M)	18:24	14:18
Subtype (D:A)	27:15	Not applicable (NA)
GDSs at sampling prior to treatment	3.00 (2.38, 4.00)	NA
WBCs in CSF (10^6^/l)	2.00 (1.00, 5.25)	NA
Protein in CSF (mg/l)	841 (649, 1285)	NA

### 3.2 Serum levels of IL-36 cytokines and other inflammatory factors in GBS and HCs

**Figure 1:**
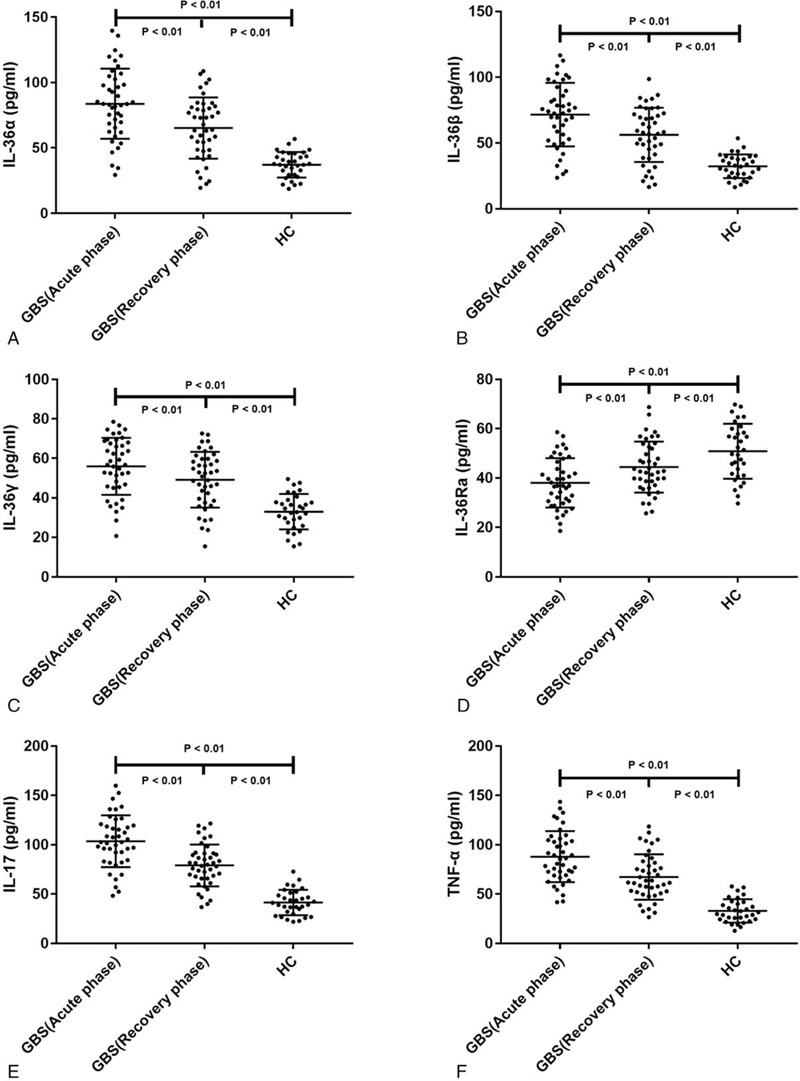
Serum IL-36 cytokines levels in the acute and recovery phase of GBS groups and in HC groups. (a) Serum IL-36α levels in the acute and recovery phase of GBS groups and in HC groups. (b) Serum IL-36β levels in the acute and recovery phase of GBS groups and in HC groups. (c) Serum IL-36γ levels in the acute and recovery phase of GBS groups and in HC groups. (d) Serum IL-36Ra levels in the acute and recovery phase of GBS groups and in HC groups. (e) Serum IL-17 levels in the acute and recovery phase of GBS groups and in HC groups. (f) Serum TNF-α levels in the acute and recovery phase of GBS groups and in HC groups.

Serum IL-36α and IL-36γ levels were significantly increased in patients with GBS during the acute phase compared to the HC group. Additionally, serum IL-36α and IL-36γ levels in the recovery phase were decreased compared to those in the acute phase, although they were still higher than those in HCs (*P* < .01, Fig. 1a and c). Meanwhile, serum IL-36β levels in the acute phase of GBS patients were slightly higher than those in HCs, while there was no significant difference between serum IL-36β levels of patients with GBS in the recovery phase and these in the acute phase or in HCs (*P* = .019, *P* = .297, and *P* = .091, respectively, Fig. 1b). In contrast, serum IL-36Ra levels were decreased in patients with GBS during the acute phase compared to the HC group. Additionally, serum IL-36Ra levels were increased in the recovery phase compared to the acute phase, but were still lower than those in HCs (*P* < .01, Fig. 1d). The serum levels of other inflammatory factors, represented by IL-17 and TNF-α, were also increased in patients with GBS during the acute phase and decreased in the recovery phase, but were still higher than those in HCs (*P* < .01, Fig. 1e and f).

### 3.3 Correlations between serum IL-36 cytokines levels and clinical parameters in GBS

**Figure 2:**
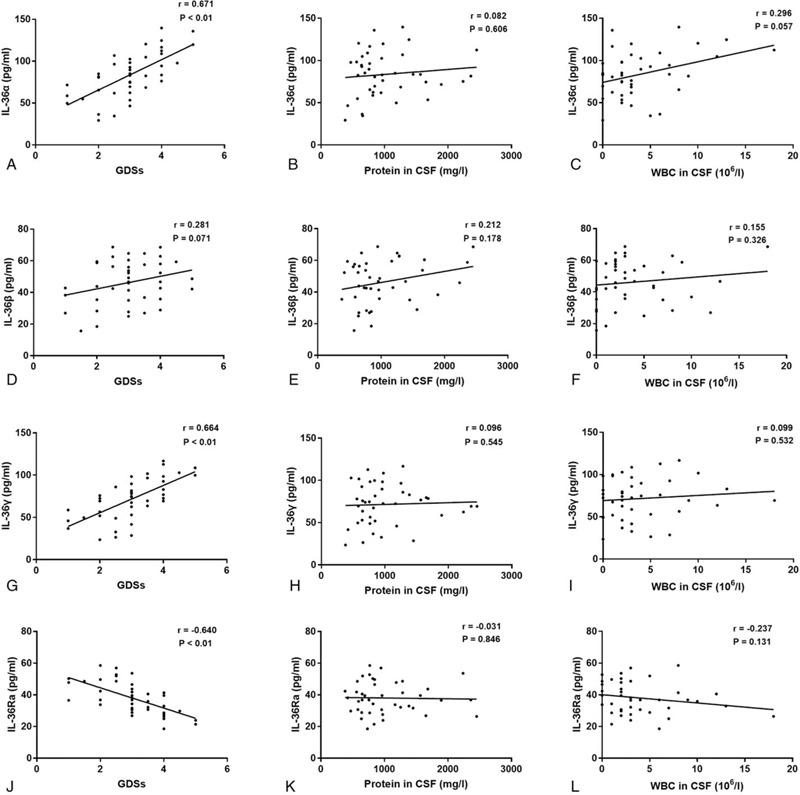
Correlations between the serum levels of IL-36 cytokines and clinical parameters in GBS. (a) The correlation between IL-36α and GDSs in GBS. (b) The correlation between IL-36α and protein in CSF in GBS. (c) The correlation between IL-36α and WBCs in CSF in GBS. (d) The correlation between IL-36β and GDSs in GBS. (e) The correlation between IL-36β and protein in CSF in GBS. (f) The correlation between IL-36β and WBCs in CSF in GBS. (g) The correlation between IL-36γ and GDSs in GBS. (h) The correlation between IL-36γ and protein in CSF in GBS. (i) The correlation between IL-36γ and WBCs in CSF in GBS. (j) The correlation between IL-36Ra and GDSs in GBS. (k) The correlation between IL-36Ra and protein in CSF in GBS. (l) The correlation between IL-36Ra and WBCs in CSF in GBS.

To evaluate the potential associations between IL-36 cytokines and GBS, we further explored the correlations between the serum levels of these cytokines and clinical parameters in patients with GBS. Our results showed that serum IL-36α and IL-36γ levels in GBS were positively correlated with GDSs (*r* = 0.671, *P* < .01, Fig. 2a; *r* = 0.664, *P* < .01, Fig. 2g, respectively), while the serum levels of IL-36Ra were negatively correlated with GDSs (*r* = −0.640, *P* < .01, Fig. 2j). Additionally, significant correlations between serum IL-36β levels and GDSs were not observed (*r* = 0.281, *P* = .071, Fig. 2d). Simultaneously, statistically significant correlations were not observed between the serum levels of different IL-36 cytokines and protein levels or white blood cell (WBC) count in the CSF of patients with GBS (*r* = 0.082, *P* = .606, Fig. 2b; *r* = 0.296, *P* = .057, Fig. 2c; *r* = 0.212, *P* = .178, Fig. 2e; *r* = 0.155, *P* = .326, Fig. 2f; *r* = 0.096, *P* = .545, Fig. 2h; *r* = 0.099, *P* = .532, Fig. 2i; *r* = −0.031, *P* = .846, Fig. 2k; *r* = −0.237, *P* = .131, Fig. 2l, respectively).

### 3.4 Correlations among the serum levels of different IL-36 cytokines in GBS

**Figure 3:**
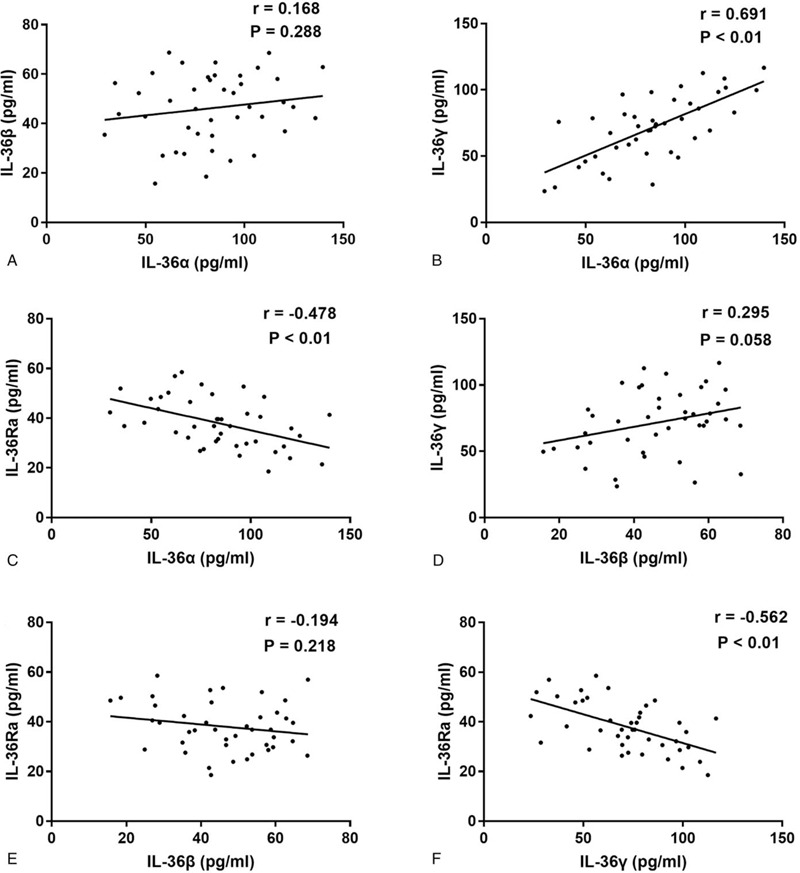
Correlations among the serum levels of different IL-36 cytokines in GBS. (a) The correlation between IL-36α and IL-36β in GBS. (b) The correlation between IL-36α and IL-36γ in GBS. (c) The correlation between IL-36α and IL-36Ra in GBS. (d) The correlation between IL-36β and IL-36γ in GBS. (e) The correlation between IL-36β and IL-36Ra in GBS. (f) The correlation between IL-36γ and IL-36Ra in GBS.

Pearson's correlation coefficients revealed negative correlations between serum IL-36α and IL-36γ levels with serum IL-36Ra levels in GBS patients (*r* = −0.478, *P* < .01, Fig. 3c; *r* = −0.562, *P* < .01, Fig. 3f, respectively). Meanwhile, serum IL-36α levels were positively correlated with serum IL-36γ levels (*r* = 0.691, *P* < .01, Fig. 3b). Additionally, statistically significant correlations were not observed between serum IL-36β levels and serum IL-36α, IL-36γ, or IL-36Ra levels in GBS patients (*r* = 0.168, *P* = .288, Fig. 3a; *r* = 0.295, *P* = .058, Fig. 3d; *r* = −0.194, *P* = .218, Fig. 3e, respectively).

### 3.5 Correlations between the serum levels of IL-36 cytokines and other inflammatory factors in GBS

**Figure 4:**
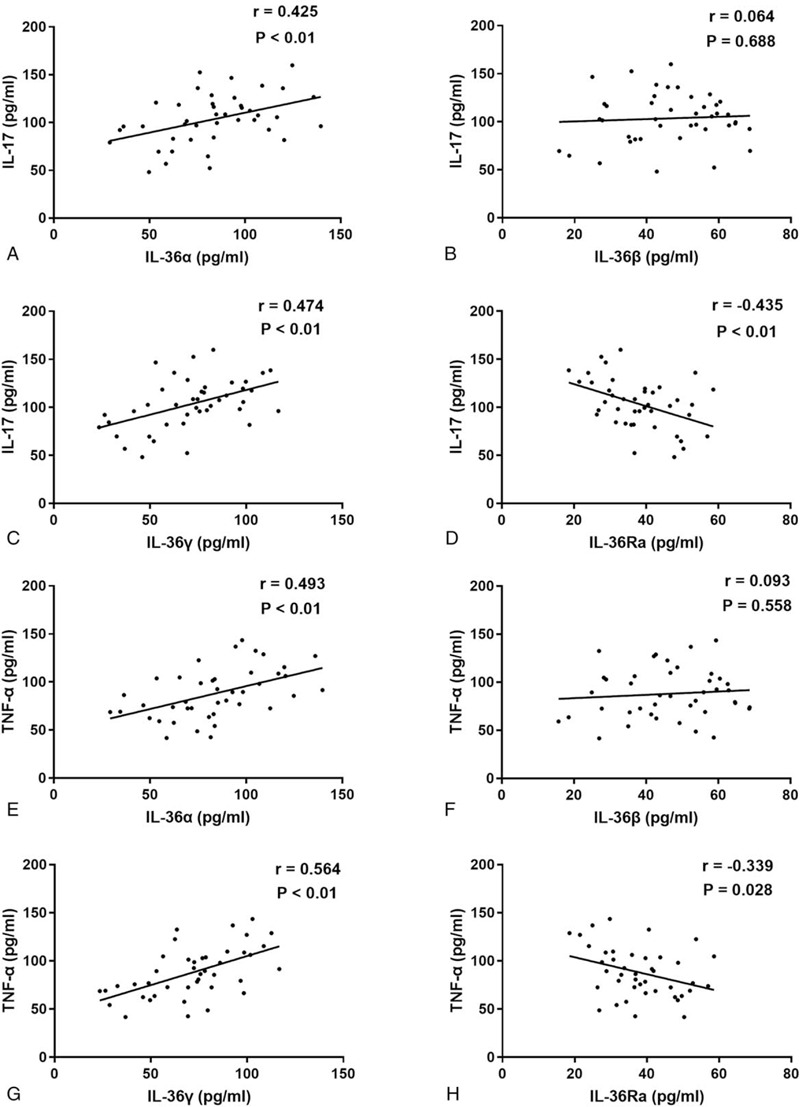
Correlations between serum levels of IL-36 cytokines and serum levels of other inflammatory factors in GBS. (a) The correlation between serum IL-36α levels and serum IL-17 levels in GBS. (b) The correlation between serum IL-36β levels and serum IL-17 levels in GBS. (c) The correlation between serum IL-36γ levels and serum IL-17 levels in GBS. (d) The correlation between serum IL-36Ra levels and serum IL-17 levels in GBS. (e) The correlation between serum IL-36α and serum TNF-α levels in GBS. (f) The correlation between serum IL-36β levels and serum TNF-α levels in GBS. (g) The correlation between serum IL-36γ levels and serum TNF-α levels in GBS. (h) The correlation between serum IL-36Ra levels and serum TNF-α levels in GBS.

To determine the possible roles of IL-36 cytokines in GBS, we further explored the correlations between the serum levels of IL-36 cytokines and other inflammatory factors, represented by IL-17 and TNF-α, which have been reported to be involved in the pathogenesis of GBS. Our results showed that the serum levels of IL-36α and IL-36γ in GBS were positively correlated with the serum levels of IL-17 or TNF-α (*r* = 0.425, *P* < .01, Fig. 4a; *r* = 0.474, *P* < .01, Fig. 4c; *r* = 0.493, *P* < .01, Fig. 4e; *r* = 0.564, *P* < .01, Fig. 4g, respectively). However, statistically significant correlations were not observed between the serum levels of IL-36β and the serum levels of IL-17 or TNF-α in GBS patients (*r* = 0.064, *P* = .688, Fig. 4b; *r* = 0.093, *P* = .558, Fig. 4f, respectively). In contrast, the serum levels of IL-36Ra were negatively correlated with the serum levels of IL-17 and TNF-α (*r* = −0.435, *P* < .01, Fig. 4d; *r* = −0.339, *P* = .028, Fig. 4h, respectively).

### 3.6 Correlations between CSF IL-36 cytokines levels and other parameters in GBS

**Figure 5:**
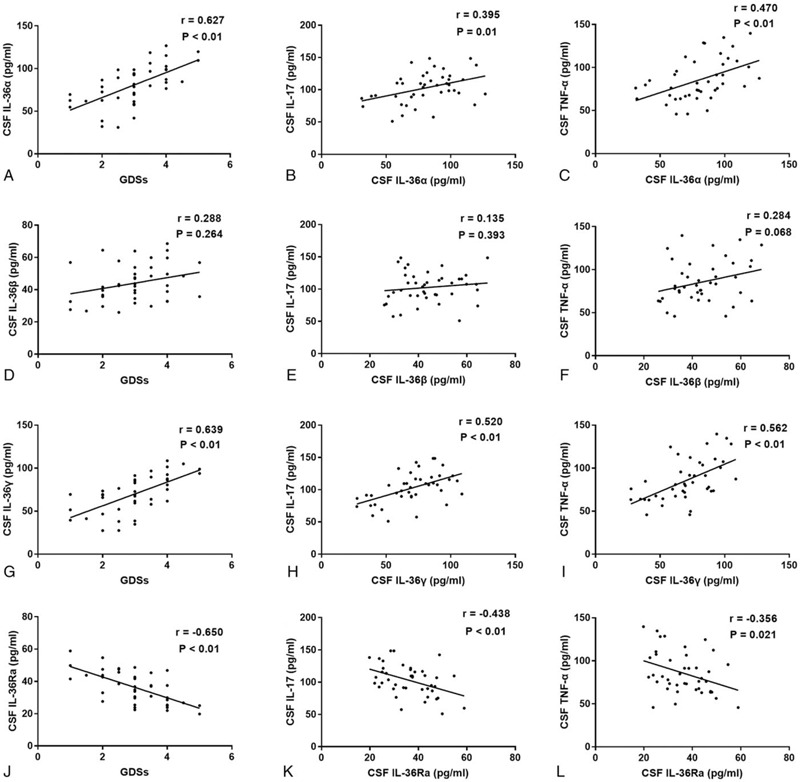
Correlations between CSF IL-36 cytokines levels and different parameters in GBS. (a) The correlation between CSF IL-36α levels and GDSs in GBS. (b) The correlation between CSF IL-36α levels and CSF IL-17 levels in GBS. (c) The correlation between CSF IL-36α levels and CSF TNF-α levels in GBS. (d) The correlation between CSF IL-36β levels and GDSs in GBS. (e) The correlation between CSF IL-36β levels and CSF IL-17 levels in GBS. (f) The correlation between CSF IL-36β levels and CSF TNF-α levels in GBS. (g) The correlation between CSF IL-36γ and GDSs in GBS. (h) The correlation between CSF IL-36γ levels and CSF IL-17 levels in GBS. (i) The correlation between CSF IL-36γ levels and CSF TNF-α levels in GBS. (j) The correlation between CSF IL-36Ra levels and GDSs in GBS. (k) The correlation between CSF IL-36Ra levels and CSF IL-17 levels in GBS. (l) The correlation between CSF IL-36Ra levels and CSF TNF-α levels in GBS.

We further explored the correlations between CSF IL-36 cytokines levels and different parameters in GBS. The results showed that CSF IL-36α and IL-36γ levels in GBS were positively correlated with GDSs (*r* = 0.627, *P* < .01, Fig. 5a; *r* = 0.639, *P* < .01, Fig. 5g, respectively), while CSF IL-36Ra levels were negatively correlated with GDSs (*r* = −0.650, *P* < .01, Fig. 5j). Additionally, significant correlations between serum IL-36β levels and GDSs were not observed (*r* = 0.288, *P* = .264, Fig. 5d). Simultaneously, statistically significant correlations were not observed between the CSF levels of different IL-36 cytokines and protein levels or white blood cell (WBC) count in the CSF of patients with GBS. Furthermore, the CSF levels of IL-36α and IL-36γ in GBS were positively correlated with the CSF levels of IL-17 and TNF-α (*r* = 0.395, *P* = .01, Fig. 5b; *r* = 0.470, *P* < .01, Fig. 5c; *r* = 0.520, *P* < .01, Fig. 5h; *r* = 0.562, *P* < .01, Fig. 5i, respectively). However, statistically significant correlations were not observed between the CSF levels of IL-36β and the serum levels of IL-17 or TNF-α in GBS patients (*r* = 0.135, *P* = .393, Fig. 5e; *r* = 0.284, *P* = .068, Fig. 5f, respectively). In contrast, the CSF levels of IL-36Ra were negatively correlated with the CSF levels of IL-17 and TNF-α (*r* = −0.438, *P* < .01, Fig. 5k; *r* = −0.356, *P* = .021, Fig. 5l, respectively).

### 3.7 Serum and CSF levels of IL-36 cytokines in different clinical subtypes of GBS

**Figure 6:**
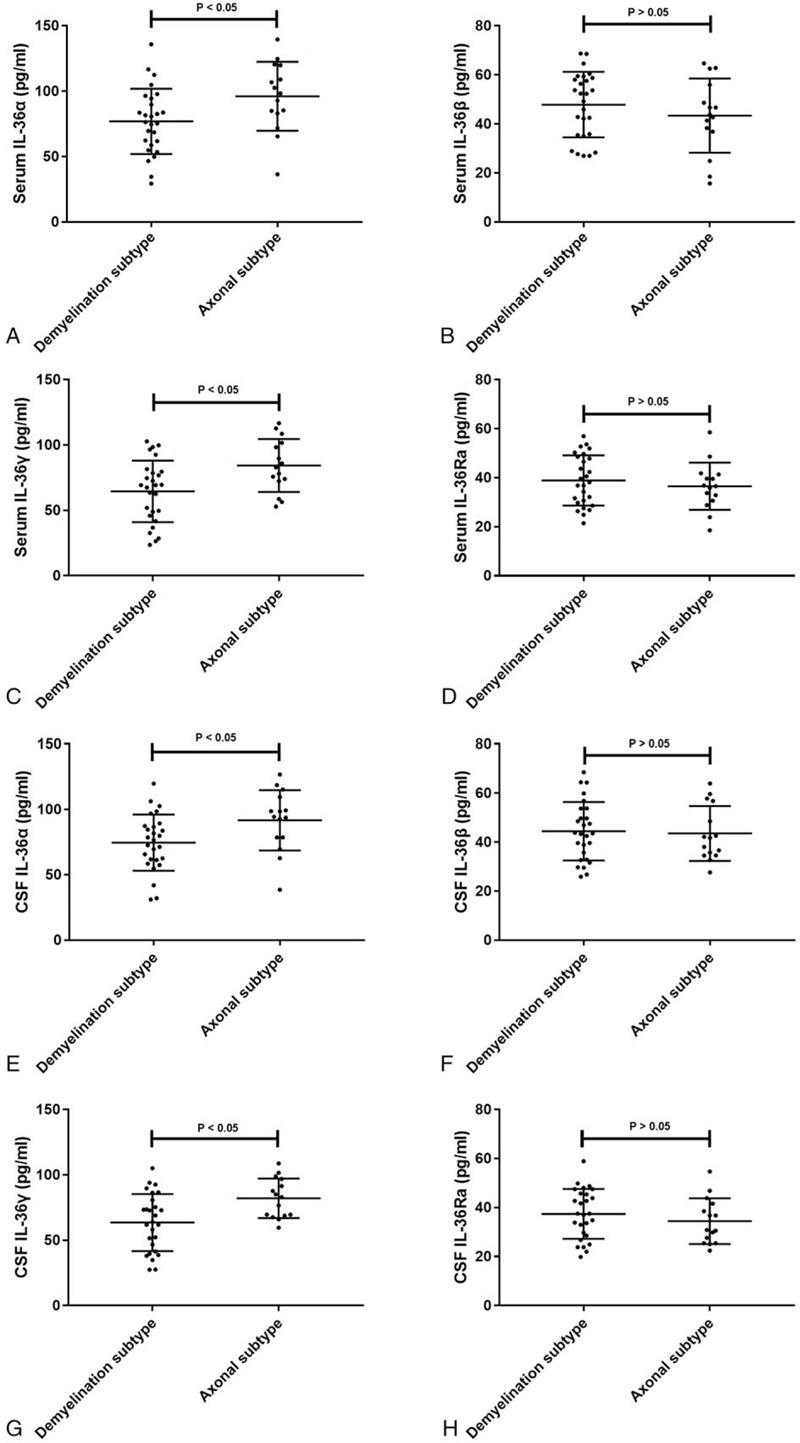
Serum IL-36 cytokines levels in different clinical subtypes of GBS. (a) Serum IL-36α levels in the demyelination subtype and axonal subtype of GBS. (b) Serum IL-36β levels in the demyelination subtype and axonal subtype of GBS. (c) Serum IL-36γ levels in the demyelination subtype and axonal subtype of GBS. (d) Serum IL-36Ra levels in the demyelination subtype and axonal subtype of GBS. (e) CSF IL-36α levels in the demyelination subtype and axonal subtype of GBS. (f) CSF IL-36β levels in the demyelination subtype and axonal subtype of GBS. (g) CSF IL-36γ levels in the demyelination subtype and axonal subtype of GBS. (h) CSF IL-36Ra levels in the demyelination subtype and axonal subtype of GBS.

To verify whether IL-36 cytokines are associated with the clinical subtype of GBS, we compared the serum levels of IL-36 cytokines in different clinical subtypes. The results showed that serum IL-36α and IL-36γ levels in patients with the axonal subtype of GBS were higher than those in patients with the demyelination subtype (*P* < .05, Fig. 6a and b). However, statistically significant differences were not observed in serum IL-36β and IL-36Ra levels between patients with different clinical subtypes of GBS (*P* > .05, Fig. 6c and d). The CSF levels of IL-36α and IL-36γ in patients with the axonal subtype of GBS were higher than those in patients with the demyelination subtype (*P* < .05, Fig. 6e and g), while significant differences in CSF IL-36β and IL-36Ra levels were not observed between patients with different clinical subtypes of GBS (*P* > .05, Fig. 6f and h).

## 4 Discussion

Cytokines and their interactions have been demonstrated to be involved in the pathogenesis of GBS.^[10,22]^ The IL-36 family is an interesting group of newly named cytokines, including IL-36α, β, γ, and IL-36Ra. IL-36 cytokines have recently been reported to be involved in the pathophysiological processes of some autoimmune diseases.^[14,23]^ However, the roles of these cytokines in GBS currently remain unclear. In this study, we performed the first analysis of the possible roles of IL-36 cytokines and their interactions in GBS.

GBS is an autoimmune neurological disorder characterized by limb weakness, weakening, or disappearance of tendon reflexes and progression over several weeks followed by gradual recovery.^[2]^ Our results showed that serum IL-36α and IL-36γ levels were significantly elevated in patients with GBS during the acute phase compared to the HC group, and these cytokines tended to decrease in the recovery phase. Conversely, serum IL-36Ra levels were decreased in patients with GBS during the acute phase compared to the HC group, but were increased in the recovery phase, although they were still lower than those in HCs. Based on these results, the altered serum levels of IL-36 cytokines may be potentially associated with the pathological processes of GBS.

IL-36 cytokines are involved in the differentiation of T helper cells 1 (Th1) and Th17 cells, and may affect the expression and secretion of related cytokines such as TNF-α, IL-17, and IL-22. Meanwhile, these secreted cytokines subsequently stimulate the expression of IL-36 family cytokines, which may form a positive feedback loop.^[24,25]^ Additionally, IL-36 cytokines also promote the maturation of dendritic cells (DCs) to further promote the differentiation of Th17 cells and the secretion of related cytokines.^[26,27]^ Th1 and Th17 cells play crucial roles in the pathogenesis of GBS, which suggests potential roles for IL-36 cytokines in GBS.^[28]^ As shown in the present study, serum IL-36α and IL-36γ levels in GBS were positively correlated with GDSs, while serum IL-36Ra levels were negatively correlated with GDSs. These results indicated that the increased serum levels of IL-36α and IL-36γ and the decreased serum IL-36Ra levels are associated with the severity of GBS. The results of correlation analyses among IL-36 cytokines revealed negative correlations between serum IL-36α and IL-36γ levels with serum IL-36Ra levels in GBS, indicating that IL-36 cytokine interactions may also be one explanation for disrupted immune balance in GBS. Some representative inflammatory cytokines, such as IL-17 and TNF-α, have been confirmed to be involved in the pathogenesis of GBS.^[10,29]^ We further explored the correlations between the levels of IL-36 cytokines and these inflammatory cytokines in GBS. Our results showed that the serum levels of IL-36α and IL-36γ in GBS were positively correlated with the serum levels of IL-17 and TNF-α in GBS patients, while the serum levels of IL-36Ra were negatively correlated with the serum levels of IL-17 and TNF-α. Additionally, statistically significant correlations were not observed between serum IL-36β levels and serum IL-17 or TNF-α levels in GBS. These results and previous evidence collectively suggested that IL-36α and IL-36γ may aggravate inflammatory injuries in GBS patients by promoting the secretion of IL-17 and TNF-α.^[30,31]^ Simultaneously, IL-17 and TNF-α may also interact to induce the expression of IL-36α and IL-36γ in GBS. Furthermore, the secretion of IL-17 and TNF-α in GBS may be mainly affected by IL-36α and IL-36γ rather than IL-36β. Recent studies have indicated that the secretion of TNF-α or IL-17 can inhibit the expression of IL-36Ra to some extent.^[30,32,33]^ Our results suggested that IL-36Ra expression may also be inhibited by TNF-α or IL-17 in GBS. Each individual member of IL-36 cytokines has been shown to exhibit a relatively specific expression pattern in different inflammatory diseases.^[34,35]^ In psoriasis and Crohn's disease, IL-36γ expression is significantly increased and suggested to be one of the major inflammatory factors, while IL-36β expression is not remarkably increased, suggesting a different cell source or mechanism regulating its expression.^[36]^ In systemic lupus erythematosus and rheumatoid arthritis, IL-36α expression is significantly up-regulated and associated plasma cells have been identified as the main producers of IL-36α.^[37,38]^ These studies and our results together suggested that IL-36 cytokines might also have distinct cell sources and expression patterns in GBS. Many pathogenic antibodies and associated immune cells play crucial roles in the occurrence and development of GBS, including plasma cells and plasmacytoid dendritic cells.^[39,40]^ Plasma cells, monocytes, and dendritic cells are the main producers of IL-36 cytokines, particularly IL-36α and IL-36γ, which may partially explain the different changes of IL-36α, IL-36γ, and IL-36β in GBS.^[35,36]^ Additionally, similar results were observed in CSF in patients with GBS. The results of correlation analyses indicated that the CSF levels of IL-36 cytokines were also associated with the severity of GBS patients. In general, the interactions among IL-36 cytokines and other inflammatory cytokines may collectively disrupt the immune balance and aggravate inflammatory injuries in GBS patients.

Clinically, GBS is broadly divided into 2 main subtypes: the demyelination subtype and the axonal subtype. The demyelination subtype mainly refers to acute inflammatory demyelinating polyneuropathy (AIDP), which is more common in Western Europe and the United States. The axonal subtypes, consist of 2 common variants: acute motor axonal neuropathy (AMAN) and acute motor and sensory axonal neuropathy (AMSAN), which are associated with prominent axonal injuries and are relatively more prevalent in China, Japan, and Mexico.^[4,5]^ Our results showed that the serum and CSF levels of IL-36α and IL-36γ in patients with the axonal subtype of GBS were higher than those in patients with the demyelination subtype, indicating that IL-36α and IL-36γ may be associated with the axonal impairment in peripheral nerves and may induce more severe inflammatory reactions in GBS. The axonal subtype of GBS is closely associated with antecedent CJ infection and the lipopolysaccharides (LPS) of CJ can induce the production of anti-GM1 and anti-GQ1b antibodies in animal models.^[3,41]^ As shown in the study by Vigne et al, LPS can significantly stimulate the expression of IL-36α and IL-36γ in bone marrow-derived DCs, while the expression of IL-36β and IL-36Ra is not remarkably increased.^[25]^ We postulate that LPS from CJ may also be involved in the pathogenesis of GBS, leading to increased serum IL-36α and IL-36γ levels. In addition, anti-ganglioside antibodies tend to be more closely associated with the axonal subtype of GBS than the demyelination subtype, and plasma cells are the indispensable source of these antibodies.^[3,42]^ Simultaneously, plasma cells are also the main producers of IL-36α and IL-36γ, which may also explain the evaluated levels of IL-36α and IL-36γ in the axonal subtype of GBS.

Of course, our study still has some limitations, including the relatively small sample sizes and lack of CSF cytokine levels measured in the control groups. Further studies can include larger sample sizes, perform additional and appropriate CSF tests, or focus on the exact mechanisms of interactions among these cytokines.

In conclusion, our study is the first to explore the potential roles of IL-36 family cytokines in GBS. Our results showed that the increased serum IL-36α and IL-36γ levels and decreased serum IL-36Ra levels are associated with the severity of GBS. Meanwhile, the results of the correlation analyses among IL-36 cytokines and other inflammatory cytokines in GBS suggested that interactions among these cytokines are potentially involved in the pathogenesis of GBS. In addition, IL-36α and IL-36γ may be more closely associated with axonal injuries in GBS. Consequently, these cytokines may not only be predictors of disease severity, but also potential markers of axonal injuries in GBS.

## Author contributions

**Conceptualization:** Zhikang Zhao, Hongbo Liu.

**Data curation:** Zhikang Zhao, Rui Zhang, Xinxin Gao, Hui Li.

**Formal analysis:** Zhikang Zhao, Hui Li.

**Investigation:** Zhikang Zhao, Rui Zhang, Xinxin Gao.

**Supervision:** Hongbo Liu.

**Writing – original draft:** Zhikang Zhao, Hui Li.

**Writing – review & editing:** Rui Zhang, Hongbo Liu.

**Conceptualization:** Zhikang Zhao, Hongbo Liu.

**Data curation:** Zhikang Zhao, Rui Zhang, Xinxin Gao, Hui Li.

**Formal analysis:** Zhikang Zhao, Hui Li.

**Investigation:** Zhikang Zhao, Rui Zhang, Xinxin Gao.

**Writing – original draft:** Zhikang Zhao, Hui Li.

**Writing – review & editing:** Hongbo Liu.

## References

[1] van Doorn PA Ruts L Jacobs BC Clinical features, pathogenesis, and treatment of Guillain-Barré syndrome. Lancet Neurol 2008;7:939–50. 18848313 10.1016/S1474-4422(08)70215-1

[2] Leonhard SE Mandarakas MR Gondim F Diagnosis and management of Guillain-Barré syndrome in ten steps. Nat Rev Neurol 2019;15:671–83. 31541214 10.1038/s41582-019-0250-9 PMC6821638

[3] Uncini A Guillain-Barré syndrome: what have we learnt during one century? A personal historical perspective. Rev Neurol (Paris) 2016;172:632–44. 27659900 10.1016/j.neurol.2016.08.006

[4] Eldar AH Chapman J Guillain Barré syndrome and other immune mediated neuropathies: diagnosis and classification. Autoimmun Rev 2014;13:525–30. 24434363 10.1016/j.autrev.2014.01.033

[5] Kuwabara S Yuki N Axonal Guillain-Barré syndrome: concepts and controversies. Lancet Neurol 2013;12:1180–8. 24229616 10.1016/S1474-4422(13)70215-1

[6] Loshaj-Shala A Colzani M Brezovska K Immunoproteomic identification of antigenic candidate *Campylobacter jejuni* and human peripheral nerve proteins involved in Guillain-Barré syndrome. J Neuroimmunol 2018;317:77–83. 29338928 10.1016/j.jneuroim.2018.01.006

[7] Rivera-Correa J de Siqueira IC Mota S Anti-ganglioside antibodies in patients with Zika virus infection-associated Guillain-Barré Syndrome in Brazil. PLoS Negl Trop Dis 2019;13:e0007695. 31527907 10.1371/journal.pntd.0007695 PMC6764688

[8] Nyati KK Prasad KN Verma A Association of TLR4 Asp299Gly and Thr399Ile polymorphisms with Guillain-Barré syndrome in Northern Indian population. J Neuroimmunol 2010;218:116–9. 19913922 10.1016/j.jneuroim.2009.10.018

[9] Goodfellow JA Willison HJ Guillain-Barré syndrome: a century of progress. Nat Rev Neurol 2016;12:723–31. 27857121 10.1038/nrneurol.2016.172

[10] Lu MO Zhu J The role of cytokines in Guillain-Barré syndrome. J Neurol 2011;258:533–48. 21104265 10.1007/s00415-010-5836-5

[11] Li S Yu M Li H IL-17 and IL-22 in cerebrospinal fluid and plasma are elevated in Guillain-Barré syndrome. Mediators Inflamm 2012;2012:260473. 23091305 10.1155/2012/260473 PMC3468147

[12] Bassoy EY Towne JE Gabay C Regulation and function of interleukin-36 cytokines. Immunol Rev 2018;281:169–78. 29247994 10.1111/imr.12610

[13] Zhou L Todorovic V Interleukin-36: structure, signaling and function. Adv Exp Med Biol 2020;20:1649. 10.1007/5584_2020_488 32026417

[14] Walsh PT Fallon PG The emergence of the IL-36 cytokine family as novel targets for inflammatory diseases. Ann N Y Acad Sci 2018;1417:23–34. 27783881 10.1111/nyas.13280

[15] Towne JE Garka KE Renshaw BR Interleukin (IL)-1F6, IL-1F8, and IL-1F9 signal through IL-1Rrp2 and IL-1RAcP to activate the pathway leading to NF-kappaB and MAPKs. J Biol Chem 2004;279:13677–88. 14734551 10.1074/jbc.M400117200

[16] Günther S Sundberg EJ Molecular determinants of agonist and antagonist signaling through the IL-36 receptor. J Immunol: official journal of the American Association of Immunologists 2014;193:921–30. 10.4049/jimmunol.1400538 24935927

[17] Gresnigt MS van de Veerdonk FL Biology of IL-36 cytokines and their role in disease. Semin Immunol 2013;25:458–65. 24355486 10.1016/j.smim.2013.11.003

[18] Schmitt V Hahn M Kästele V Interleukin-36 receptor mediates the crosstalk between plasma cells and synovial fibroblasts. Eur J Immunol 2017;47:2101–12. 28857172 10.1002/eji.201646788

[19] Dinarello CA The IL-1 family of cytokines and receptors in rheumatic diseases. Nat Rev Rheumatol 2019;15:612–32. 31515542 10.1038/s41584-019-0277-8

[20] Madonna S Girolomoni G Dinarello CA The significance of IL-36 hyperactivation and IL-36R targeting in psoriasis. Int J Mol Sci 2019;20. 10.3390/ijms20133318 PMC6650959 31284527

[21] Wang S Liu Y Nie M Profile of IL-36 cytokines (IL-36α, IL-36β, IL-36γ and IL-36Ra) in patients with primary immune thrombocytopenia. Int Immunopharmacol 2020;82:106341. 32114410 10.1016/j.intimp.2020.106341

[22] Peng J Zhang H Liu P IL-23 and IL-27 levels in serum are associated with the process and the recovery of Guillain-Barré syndrome. Sci Rep 2018;8:2824. 29434217 10.1038/s41598-018-21025-5 PMC5809385

[23] Queen D Ediriweera C Liu L Function and regulation of IL-36 signaling in inflammatory diseases and cancer development. Front cell Dev Biol 2019;7:317. 31867327 10.3389/fcell.2019.00317 PMC6904269

[24] Gresnigt MS Rösler B Jacobs CW The IL-36 receptor pathway regulates Aspergillus fumigatus-induced Th1 and Th17 responses. Eur J Immunol 2013;43:416–26. 23147407 10.1002/eji.201242711

[25] Vigne S Palmer G Lamacchia C IL-36R ligands are potent regulators of dendritic and T cells. Blood 2011;118:5813–23. 21860022 10.1182/blood-2011-05-356873

[26] Mutamba S Allison A Mahida Y Expression of IL-1Rrp2 by human myelomonocytic cells is unique to DCs and facilitates DC maturation by IL-1F8 and IL-1F9. Eur J Immunol 2012;42:607–17. 22144259 10.1002/eji.201142035

[27] Tortola L Rosenwald E Abel B Psoriasiform dermatitis is driven by IL-36-mediated DC-keratinocyte crosstalk. J Clin Invest 2012;122:3965–76. 23064362 10.1172/JCI63451 PMC3484446

[28] Chen X Guo Y Han R Class I PI3K inhibitor ZSTK474 attenuates experimental autoimmune neuritis by decreasing the frequency of Th1/Th17 cells and reducing the production of proinflammatory cytokines. Cell Immunol 2018;329:41–9. 29724464 10.1016/j.cellimm.2018.04.011

[29] Han R Xiao J Zhai H Dimethyl fumarate attenuates experimental autoimmune neuritis through the nuclear factor erythroid-derived 2-related factor 2/hemoxygenase-1 pathway by altering the balance of M1/M2 macrophages. J Neuroinflammation 2016;13:97. 27142843 10.1186/s12974-016-0559-x PMC4855950

[30] Furue K Yamamura K Tsuji G Highlighting interleukin-36 signalling in plaque psoriasis and pustular psoriasis. Acta Derm Venereol 2018;98:5–13. 28967976 10.2340/00015555-2808

[31] Żebrowska A Woźniacka A Juczyńska K Correlation between IL36α and IL17 and activity of the disease in selected autoimmune blistering diseases. Mediators Inflamm 2017;2017:8980534. 28611508 10.1155/2017/8980534 PMC5458385

[32] Do MS Jeong HS Choi BH Inflammatory gene expression patterns revealed by DNA microarray analysis in TNF-alpha-treated SGBS human adipocytes. Yonsei Med J 2006;47:729–36. 17066518 10.3349/ymj.2006.47.5.729 PMC2687760

[33] Mercurio L Morelli M Scarponi C IL-38 has an anti-inflammatory action in psoriasis and its expression correlates with disease severity and therapeutic response to anti-IL-17A treatment. Cell Death Dis 2018;9:1104. 30377293 10.1038/s41419-018-1143-3 PMC6207563

[34] Murrieta-Coxca JM Rodríguez-Martínez S Cancino-Diaz ME IL-36 cytokines: regulators of inflammatory responses and their emerging role in immunology of reproduction. Int J Mol Sci 2019;20:1649. 10.3390/ijms20071649 PMC6479377 30987081

[35] Buhl AL Wenzel J Interleukin-36 in infectious and inflammatory skin diseases. Front Immunol 2019;10:1162. 31191535 10.3389/fimmu.2019.01162 PMC6545975

[36] Boutet MA Bart G Penhoat M Distinct expression of interleukin (IL)-36α, β and γ, their antagonist IL-36Ra and IL-38 in psoriasis, rheumatoid arthritis and Crohn's disease. Clin Exp Immunol 2016;184:159–73. 26701127 10.1111/cei.12761 PMC4837235

[37] Chu M Wong CK Cai Z Elevated expression and pro-inflammatory activity of IL-36 in patients with systemic lupus erythematosus. Molecules 2015;20:19588–604. 26516833 10.3390/molecules201019588 PMC6332178

[38] Frey S Derer A Messbacher ME The novel cytokine interleukin-36α is expressed in psoriatic and rheumatoid arthritis synovium. Ann Rheum Dis 2013;72:1569–74. 23268368 10.1136/annrheumdis-2012-202264

[39] Naik GS Meena AK Reddy B Anti-ganglioside antibodies profile in Guillain-Barré syndrome: correlation with clinical features, electrophysiological pattern, and outcome. Neurol India 2017;65:1001–5. 28879885 10.4103/neuroindia.NI_1226_15

[40] Wang YZ Feng XG Wang Q Increased plasmacytoid dendritic cells in Guillain-Barré syndrome. J Neuroimmunol 2015;283:1–6. 26004148 10.1016/j.jneuroim.2015.03.019

[41] Ang CW De Klerk MA Endtz HP Guillain-Barré syndrome- and Miller Fisher syndrome-associated *Campylobacter jejuni* lipopolysaccharides induce anti-GM1 and anti-GQ1b antibodies in rabbits. Infect Immun 2001;69:2462–9. 11254608 10.1128/IAI.69.4.2462-2469.2001 PMC98180

[42] Nutt SL Hodgkin PD Tarlinton DM The generation of antibody-secreting plasma cells. Nat Rev Immunol 2015;15:160–71. 25698678 10.1038/nri3795

